# Advances in Cardiovascular Disease Lipid Research Can Provide Novel Insights Into Mycobacterial Pathogenesis

**DOI:** 10.3389/fcimb.2019.00116

**Published:** 2019-04-18

**Authors:** Shyamala Thirunavukkarasu, Shabaana A. Khader

**Affiliations:** Department of Molecular Microbiology, Washington University in St. Louis School of Medicine, St. Louis, MO, United States

**Keywords:** mycobacterium, tuberculosis, granuloma, lipid, cardiovascular, plaque, arachidonic acid, foam cell

## Abstract

Cardiovascular disease (CVD) is the leading cause of death in industrialized nations and an emerging health problem in the developing world. Systemic inflammatory processes associated with alterations in lipid metabolism are a major contributing factor that mediates the development of CVDs, especially atherosclerosis. Therefore, the pathways promoting alterations in lipid metabolism and the interplay between varying cellular types, signaling agents, and effector molecules have been well-studied. Mycobacterial species are the causative agents of various infectious diseases in both humans and animals. Modulation of host lipid metabolism by mycobacteria plays a prominent role in its survival strategy within the host as well as in disease pathogenesis. However, there are still several knowledge gaps in the mechanistic understanding of how mycobacteria can alter host lipid metabolism. Considering the in-depth research available in the area of cardiovascular research, this review presents an overview of the parallel areas of research in host lipid-mediated immunological changes that might be extrapolated and explored to understand the underlying basis of mycobacterial pathogenesis.

## Introduction

One of the leading causes of morbidity and mortality in westernized countries is cardiovascular disease (CVD), such as atherosclerosis (Yeates et al., 2015). Atherosclerosis is a complex, chronic, progressive, inflammatory disease involving different cell types resulting in the formation of an atheromatous plaque. Atherosclerosis is characterized by infiltration of the arterial intima by macrophages which scavenge oxidized low density lipoprotein (oxLDL), which further promotes alterations in cholesterol influx, esterification and efflux, ultimately resulting in the progression of the macrophage into a foam cell. The specific contributions of lipids and lipoproteins as well as the influence of cholesterol metabolism in the formation of atheromatous plaques has been extensively researched in the context of atherosclerosis and CVD (Chroni et al., 2011).

*Mycobacterium tuberculosis* (*Mtb*) is a successful human pathogen due to its ability to cause tuberculosis (TB) in almost 10 million individuals annually (Dye and Williams, 2010). Non-tuberculous mycobacteria (NTM) also cause diseases such as pulmonary and skin infections, in addition to being implicated as putative causative agents of Crohn's disease in humans (Thirunavukkarasu et al., 2017a). NTM share commonalities with tuberculous mycobacteria with regard to subversion of host macrophage immune responses (Whittington et al., 2012; Thirunavukkarasu et al., 2017a). A primary reason for the ubiquitous spread of mycobacterial infection despite current control strategies is the ability of pathogenic mycobacteria to persist in a non-replicative state both within the host, and sometimes in the environment (Falkinham, 2009). Macrophages play a pivotal role in the immune response against mycobacteria (Pieters, 2008; Thirunavukkarasu et al., 2015; Mcclean and Tobin, 2016). One of the main mechanisms of the successful intra-macrophage survival of mycobacteria including *Mtb, M. avium, M. bovis, M. paratuberculosis, M. ulcerans*, and *M. leprae* is their capacity to manipulate the host cellular metabolism to utilize intracellular substrates including fatty acids and cholesterol (Mendum et al., 2015). This manipulation of the host macrophage lipid metabolic pathway is a hallmark of several mycobacterial infections including TB (Peyron et al., 2008; Russell et al., 2009; Almeida et al., 2012; Caire-Brändli et al., 2014). Dysregulated lipid metabolism resulting in foam cell formation in macrophages and other cell types, and its association with steroid hormones as well as granuloma lesion formation, is a critical aspect in understanding mycobacterial pathogenesis. However, current research on the contribution of host lipid metabolic pathways in disease pathogenesis is limited, unlike in cardiovascular research where it has been the focus of extensive studies (Tambo et al., 2016). The similarities in the immune responses in the kinetics of atherosclerotic plaque formation and a granuloma formation in TB is an exposition of how knowledge could be gained by extrapolating ideas from among these fields.

NIH has identified interdisciplinarity as an essential contributor to needed knowledge and made it an explicit priority in its roadmap. Considering the several areas of similarities between the immunopathology of atherosclerosis and mycobacteriosis, it would be applicable to explore and extrapolate the plethora of information available in this arena in CVD research to address the knowledge gaps in the area of host lipid metabolism in mycobacterial research. However, comprehensive review articles providing reference pools for promoting scientific knowledge in interdisciplinary applications between CVD and mycobacterial immunopathology are lacking. Therefore, the purpose of this review is to identify and put forth the similarities in relation to alterations in host lipid metabolism contributing to disease pathology induced by cardiovascular and mycobacterial diseases. Furthermore, we highlight the recent advances pertaining to host lipid metabolism in CVD immunopathology that could provide potential avenues to explore for researchers involved in studying mycobacterial pathogenesis.

## Macrophage Intracellular Lipid Metabolism and Foam Cell Formation

A key feature in atherosclerosis and mycobacteriosis is the presence of lipid laden cells called foam cells (Kruth, 2001; Chen et al., 2008; Almeida et al., 2012; Bah et al., 2017). The process of foam cell formation is directly or indirectly influenced by uptake of native or modified lipoproteins by cell types present in atheromatous lesions especially macrophages, subsequent processing and retention of intracellular lipids, and altered reverse cholesterol transport (Remmerie and Scott, 2018). Lipids are not soluble in plasma and circulating lipids are transported to various tissues in the form of lipoproteins. Additionally, lipids can also circulate in blood in an albumin bound manner and can be taken into macrophages via micropinocytosis in a receptor independent manner (Kruth, 2013).

Lipoproteins are complex particles made up of a central hydrophobic core of non-polar lipids, primarily cholesterol esters, and triacyl glycerides (TAG). This hydrophobic core is surrounded by a hydrophilic membrane consisting of phospholipids, free cholesterol, and apolipoproteins (Chistiakov et al., 2017). Based on size and concentration, lipoproteins are classified as chylomicrons, very low density lipoproteins (VLDL), low density lipoproteins (LDL), or high density lipoproteins (HDL). LDL particles transport cholesterol esters, by associating with apolipoproteins B-100 and C-III, and are taken up inside the cell via the low density lipoprotein receptor (LDLR) (Rosenson et al., 2011). Once inside, LDL is digested inside lysosomes and free cholesterol is released. Free cholesterol can then either move to the membrane surface and undergo efflux via ATP-binding cassette transporter (ABC) A1 or ABCG1, or enter the endoplasmic reticulum (ER) where they are esterified to form lipid droplets by acyl CoA acetyl transferase 1 (ACAT-1) (Chistiakov et al., 2017). Free cholesterol could also cause peristent stress to the ER which ultimately results in excess nuclear factor kappa B (NFκB) signaling and apoptosis. Excess free cholesterol inside the cells can also result in the formation of cholesterol crystals, leading to enrichment of the cell membrane lipid rafts causing toll-like receptor (TLR) activation, and downstream signaling events (Remmerie and Scott, 2018).

**Figure 1:**
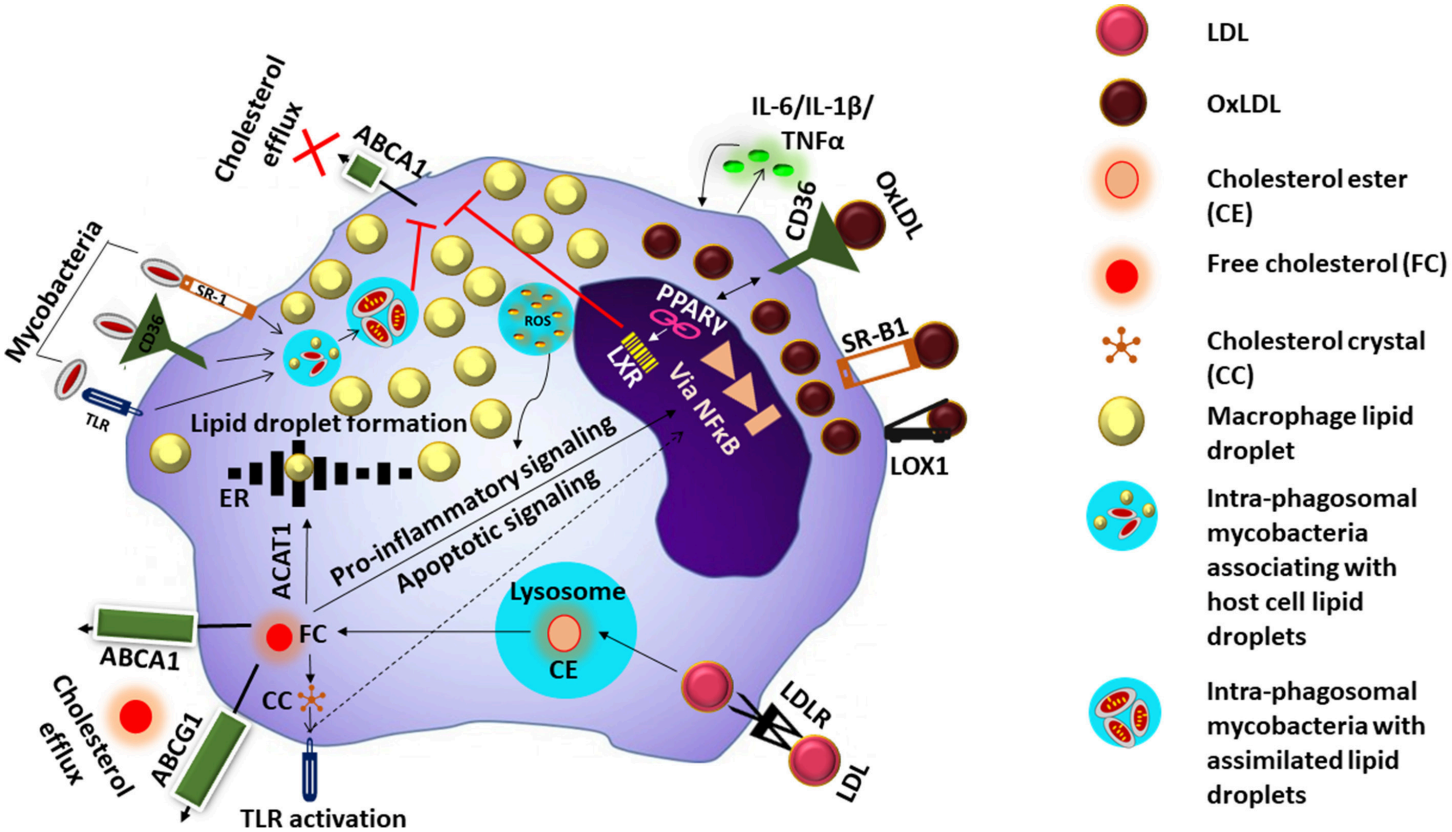
Mechanism of foam cell formation. LDL is taken up inside the cell via the low density lipoprotein receptor (LDLR). Digestion of the cholesterol esters (CE) in LDL inside the lysosome releases free cholesterol (FC) which undergoes efflux via ATP-binding cassette transporter (ABC) A1 or ABCG1, or enter the endoplasmic reticulum (ER) where they are esterified to form lipid droplets by acyl CoA acetyl transferase 1 (ACAT-1) FC could also cause persistent stress to the ER which ultimately results in excess nuclear factor kappa B (NFkB) signaling and apoptosis. Excess FC inside the cells can also result in the formation of cholesterol crystals (CC) causing toll-like receptor (TLR) activation and downstream signaling events. OxLDL is taken up via scavenger receptors such as CD36, scavenger receptor B1 (SRB1) and LOX1, and induces ROS generation and cytokine secretion. Components of oxLDL are ligands for the peroxisome proliferator activated receptor gamma (PPARγ) which increases the intake of more OxLDL via upregulation of CD36. Activation of PPARγ triggers liver X receptor signaling which facilitates lipid efflux via upregulation of ABCAI, blocking of this pathway along with accumulation of OxLDL/FC results in the macrophage transforming into a foam cell. Mycobacteria entering the cell via the cell surface receptors are taken up by phagosomes and associate with lipid droplets and assimilate them facilitating their survival in the host cell whose lipid metabolism is further impacted by the mycobacteria.

LDL also undergoes oxidation resulting in the formation OxLDL. The sub-endothelial space is considered to be the site of LDL oxidation as the antioxidant properties of blood prevents LDL oxidation while in circulation (Matsuura et al., 2008). LDL modification to OxLDL occurs by multiple mechanisms including oxidative and nitrosative stress (ROS and NOS) as well as enzymes like 12/15 lipoxygenases (LOX) and secretory phospholipase A2 (sPLA2) (Linton et al., 2015). OxLDL comprises of a variety of by-products arising due to the modification of both lipids and apolipoprotein B by lipid peroxidation (Matsuura et al., 2008). OxLDL is taken up preferentially by the macrophages by endocytosis, via scavenger receptors such as CD36, macrophage receptor with collagenous structure (MARCO), scavenger receptor B1 (SR-B1), and LOX1, as well as CD14-Toll like receptor 4-MD2 complex (Kruth, 2001). This induces ROS generation, cytokine secretion, production of myeloperoxidase, and 12/15 lipoxygenase secretion which further oxidize new LDL thus increasing the local pool of oxLDL. The components of oxLDL also act as ligands for the peroxisome proliferator activated receptor gamma (PPARγ) which in turn increases the intake of more OxLDL via upregulation of CD36. Moreover, accumulating oxLDL blocks the PPARγ mediated liver X receptor (LXR) signaling which favors cholesterol efflux due to upregulation of ABCA1. The events result in the macrophage transforming into a foam cell (Figure 1). Internalization of OxLDL by CD36 decreases macrophage migration and activates focal adhesion kinases. Therefore, OxLDL contributes to both macrophage activation as well as retention (Parthasarathy et al., 2010). In addition, OxLDL increases platelet derived growth factor (PDGF) and other growth factors secretion thus amplifying cellular proliferation. The OxLDL mediated ROS generated triggers the cytochrome c mediated apoptosis cascade leading to cellular death and debris accumulation (Leiva et al., 2015). This coupled with a malfunctioning lipid metabolic pathway can impact the apoptotic cell clearance by other macrophages resulting in chronic inflammation (Szondy et al., 2014), which may further enhance foam cell formation. *Mtb* which enter the host macrophages has the capacity to utilize host cholesterol and also sequester host fatty acids in the form of TAG within intra-bacterial lipid inclusion bodies believed to occur via bacterial *mce* transporters mediated by *Rv3723/LucA* (Pandey and Sassetti, 2008; Daniel et al., 2011; Nazarova et al., 2017). This lipid accquisition is required for bacterial persistence especially in the chronic phase of disease (Neyrolles, 2014).

Since foam cell formation is an essential aspect of the immunopathology of both atherosclerosis and several mycobacterial infections, the knowledge regarding the lipid mediators, and signaling events that contribute to this are of interest.

### Arachidonic Acid and Lipid Mediators

**Figure 2:**
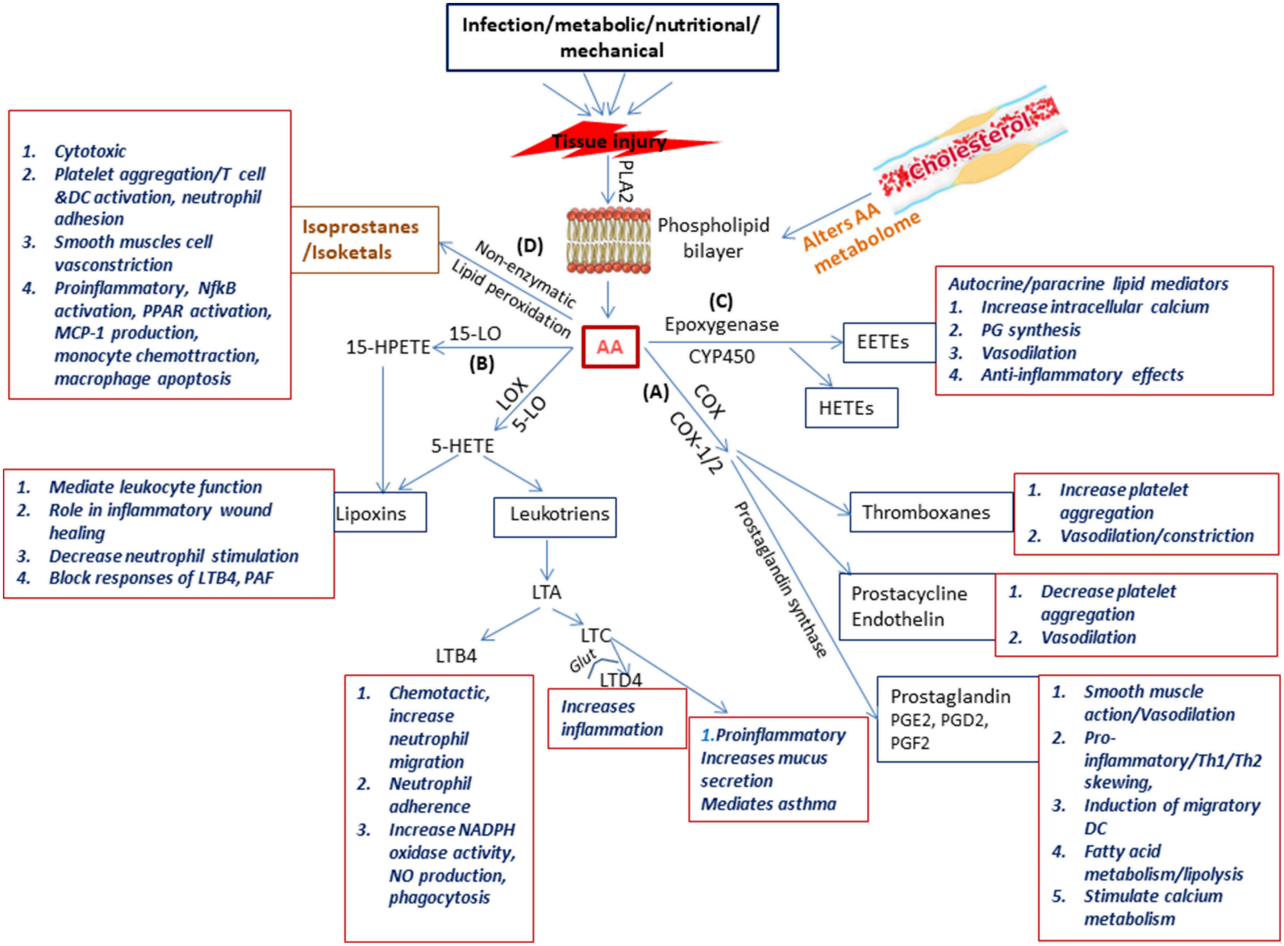
Schematic representation of the physiological generation and function of lipid mediators in immunity. Injury to the tissue due to infection, nutritional, metabolic, or mechanical causes results on secretion of phospholipase A2 which acts on the phospholipid layer of the cell membrane generating arachidonic acid (AA). Arachidonic acid is the substrate from which the biologically active lipid mediators belonging to the eicosanoid family are produced due to the activity of enzymes belonging to three families **(A)** the COX pathway (COX1 COX2) resulting in production of prostaglandins **(B)** the LOX pathway (5-LOX, 1LOX, 15LOX) resulting in production of leukotriens and lipoxins, and **(C)** the cytochrome p450 pathway which forms the hydroxyeicosotetranoic acids and the epoxyeicosotrienoic acids. In addition **(D)** Non-enzymatic lipid peroxidation of AA results in the production of isoprostanes and isoketals. All these lipd mediators have a wide range of biologically significant functions which impact immunity.

OxLDL activates phospholipase enzymes which act on membrane phospholipids releasing arachidonic acid (AA) (Akiba et al., 2003). Arachidonic acid is the substrate from which the biologically active lipid mediators belonging to the eicosanoid family are produced due to the activity of various enzymes (Akiba et al., 2003). These enzymes belong to three pathways, namely (a) the COX pathway (COX1, COX2) resulting in production of prostaglandins (PG), (b) the LOX pathway (5-LOX, 1LOX, 15LOX) resulting in production of leukotrienes (LTs) and lipoxins (LXs), and (c) the cytochrome P450 (Cyp) pathway which forms the hydroxyeicosotetranoic acids (HETEs) and the epoxyeicosotrienoic acids (EETEs) (Hanna and Hafez, 2018). Non-enzymatic lipid peroxidation of AA results in the production of isoprostanes and isoketals. PGs and LTs are the initial mediators which recruit neutrophils and monocytes to the site of inflammation (Dietzold et al., 2015). The importance and involvement of PGE2 and other lipid mediators and the signaling events of the arachidonic acid metabolic pathway (Figure 2) in CVDs in general and atherosclerosis in particular has been extensively explored utilizing several approaches which could offer insight for mycobacterial researchers to pursue.

## Lesion: Atherosclerotic Plaque vs. TB Granuloma

An atherosclerotic plaque formed as a result of dysregulated lipid metabolism is either stable and characterized by low inflammatory cell infiltration with a thick fibrous cap or an unstable plaque characterized by a necrotic core of foamy macrophages, degradation of the collagenous fibrous cap by matrix metalloproteinases, and subsequent rupture of the plaque necrotic core (Kruth, 2001). Several enzymes including those belonging to the Cyp family play crucial roles in the generation of either a stable plaque or progression to an unstable plaque with poor prognosis for atherosclerosis outcome (Silvestre-Roig et al., 2014; Song et al., 2016; Stefanadis et al., 2017).

**Figure 3:**
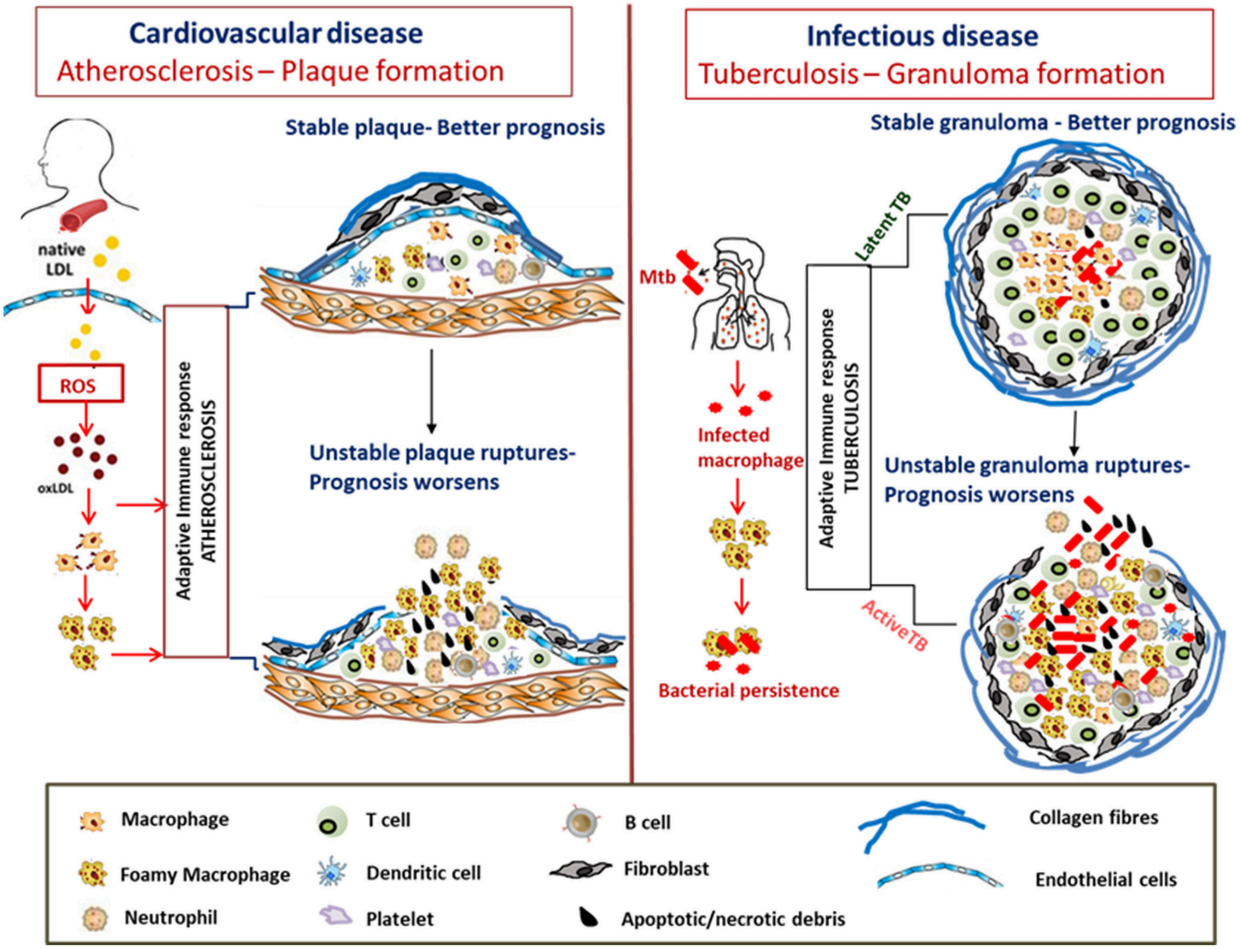
Similarities in formation and progression of atherosclerotic plaques and tuberculous granulomas. In atherosclerosis, LDLs accumulate in the arterial intima, undergo modification by oxidation to form oxLDL which act as chronic stimulators of the innate and adaptive immune response. OxLDL is taken up by the macrophages via scavenger receptors forming foam cells. The plaque is composed of neutrophils, platelets, T cells, and DC along with foam cells and resultant secretion of pro-inflammatory cytokines that accentuate inflammation. Apoptosis and secondary necrosis of foam cells and SMCs result in necrotic core development, degradation of the collagen layer forming unstable plaque that eventually ruptures resulting in bad prognosis. *Mtb* infection is also characterized by foam cell formation which is crucial for bacterial peristence. The cellular composition of TB granulomatous lesions includes infected and uninfected macrophages, foamy macrophages, B and T lymphocytes, neutrophils, platelets, T cells, and fibroblasts. The production of inflammatory cytokines and necrosis of the lipid laden foam cells at the granulomatous core result in disorganization of the granuloma favoring dissemination of bacteria worsening the prognosis.

The stability of the atheromatous plaques formed directs disease outcome in atherosclerosis (Chen et al., 2016). Similarly, many mycobacterial infections including *Mtb, M. paratuberculosis, M. bovis*, and *M. marinum* are characterized by granuloma formation, the relative stability and organization of which impacts disease severity and outcome (Wangoo et al., 2005; Silva Miranda et al., 2012; Wu et al., 2012; Guirado and Schlesinger, 2013; Fernández et al., 2016). Granulomas are formed as organized aggregates of immune cells composed primarily of infected and uninfected macrophages, foamy macrophages, and other cell types including NK cells and DCs that are surrounded by a ring of B and T lymphocytes (Silva Miranda et al., 2012). Granulomas are formed as a means to control chronic mycobacterial infection, but progression to cavitary granuloma leads to subclinical and clinical disease (Guirado and Schlesinger, 2013). Thus, the TB granuloma is categorized as either stable wherein the bacteria are contained and infection is mostly latent or as a disorganized granuloma with a necrotic caseous core, matrix metalloproteinase-mediated loss of the collagen layer, and dissemination of infection leading to active TB with a much poorer prognosis (Kim et al., 2010; Russell et al., 2010). Although there are differences between an atherosclerotic plaque and TB granuloma regarding the role of T cells and B cells, similarities in dysregulated host lipid metabolism which contribute to disease pathogenesis are indeed conspicuous in both diseases (Figure 3).

Cholesterol catabolism, apart from providing a potent source of carbon for mycobacterial metabolism (Wilburn et al., 2018), also results in the production of propionyl CoA and methylmalonyl CoA that are used for the synthesis of mycobacterial cell wall virulence components such as phthiocerol dimycocerosate (PDIM) (Jain et al., 2007), and sulpholipids (Pandey and Sassetti, 2008; Griffin et al., 2012; Abuhammad, 2017). Since cholesterol modulates mycobacterial metabolism, identification of novel targets in the cholesterol metabolic pathway has been carried out for the purpose of chemotherapeutic intervention of TB (Vanderven et al., 2015). Moreover, the modulation of host cholesterol can have adverse effects on several host signaling mechanisms as well as cellular immune polarization. It is believed that lipid mediated signaling via the sterol regulatory element binding protein 1 leads to a pro-inflammatory M1 phenotype in macrophages while signaling via LXR leads to an anti-inflammatory M2 phenotype (Tangirala et al., 2002). Cholesterol also acts as a substrate for various enzyme activities including those belonging to the Cyp family (Pikuleva, 2006; Rogers et al., 2015). Furthermore, cholesterol is also a precursor for major sex hormones and a role for sex differences in the host immune responses to CVD (Jennings et al., 2014; Pingili et al., 2015) and mycobacterial infections (Neyrolles and Quintana-Murci, 2009; Bini et al., 2014; Karunasena et al., 2014; Nhamoyebonde and Leslie, 2014; Mirsaeidi and Sadikot, 2015; Dibbern et al., 2017), has been of interest in recent years, with men reportedly being more susceptible than women to CVD as well as mycobacterial infection. In this context, it is important to note that although a role for sex steroid hormones in influencing susceptibility to pulmonary tuberculosis has also been established (Bini et al., 2014), it has not been researched indepth as is the case in CVD.

## Role of Lipid Metabolism in Mycobacterial Pathogenesis

The outcome of initial exposure of *Mtb* in the human host could either result in the pathogen progressing to primary active TB disease or efficient elimination through an innate and/or acquired immune response. Mycobacteria could also survive inside the host in a non-replicative state but capable of reactivation and subsequent progression to the active form of disease. The active phase of TB infection is characterized by the formation of caseous granulomas and cavities which are correlated with failure of control and disease relapse (Russell et al., 2010). The progression of human TB infection, from latent infection to active disease, occurs as a continuous spectrum of metabolic bacterial activity amidst the onslaught of protective host immune responses (Drain et al., 2018). Furthermore, the asymptomatic but well-contained disease forms in the early stages of infection are classified as incipient disease while the asymptomatic disease at the latent end of the spectrum associated with loss of effective containment is termed as subclinical TB (Achkar and Jenny-Avital, 2011). Several studies have reported on the role of host and pathogen derived lipids both *in vitro* and *in vivo* in disease pathogenicity caused by mycobacterial sp. in general, and *Mtb* in particular (Guenin-Mace et al., 2009; Philips and Ernst, 2012; Lee et al., 2013; Thirunavukkarasu et al., 2014, 2016a). Thus, the modulation of the host lipid metabolism could have an impact on the different stages of the TB disease spectrum, including granuloma formation. In the following sections we provide a snapshot of the lipid mediators and pathways that affect TB disease progression and outcome.

### Role of Lipid Mediators in TB Progression

Foamy macrophages formed during the course of mycobacterial infection are rich sites of PGE2 generation (Almeida et al., 2009) and therefore cross regulation of eicosanoid pathways could play a critical role in controlling the growth of pathogenic mycobacteria. PGE2 is an important lipid mediator as it has the potential to regulate its own production, while suppressing acute inflammatory mediators, resulting in its predominance at late/chronic stages of immunity during a wide range of autoimmune, metabolic, and infectious diseases (Kalinski, 2012). Therefore, inhibition of PGE2 production and signaling could potentially represent a therapeutic alternative to treat bacterial infections in the chronic phase (Agard et al., 2013), although augmentation of PGE2 levels reportedly improves protection against *Mtb* in the acute stages of infection (Mayer-Barber et al., 2014). Apart from PGE2, another area gaining importance in mycobacterial research in recent years is the lipoxygenase pathway. Mice deficient in 5-LOX were found to be more resistant to *Mtb* infection (Bafica et al., 2005; Chen et al., 2008). Moreover, LOX-derived eicosanoids are known to regulate DC maturation and function as well as a neutrophilic recruitment cascade during *Mtb* infection (Rothe et al., 2015; Mishra and Sassetti, 2016). Zebra fish larvae deficient in LTA4 hydrolase were found to have enhanced susceptibility to *M. marinum* during the chronic phase of infection, due to the utilization of eicosanoid substrates leading to the production of anti-inflammatory lipoxins and an increase in TNF-α levels (Tobin et al., 2010, 2012). Hence, it is likely that these pathways/mediators might have a differential effect depending on the stage of infection which could in turn define the final disease outcome.

Inhibition of apotosis is a mechanism by which mycobacteria can persist in the host and this is also reported to occur at the level of lipid mediators (Dietzold et al., 2015). Virulent strains of *Mtb* are known to enhance production of leukotriens while suppressing cyclooxygenase (COX)2-induced PGE2 synthesis, which drives the cells toward necrosis (Rocca and Fitzgerald, 2002; Chen et al., 2008). Moreover, it is known that *Mtb* also utilizes host ceramide, which reduces the capacity of aveolar macrophages to clear apoptotic cells leading to persistent inflammation (Mattos et al., 2011). Of interest, ceramides are a known antagonist of foam cell formation via decreased transport of CD36 to the macrophage cell membrane (Chistiakov et al., 2016), therefore utilizing the host ceramide supply could indirectly drive foam cell formation and disease progression.

### Cytokines Involved in Modulating Host Lipid Metabolism in *Mtb* Infection

Mycobacterial lipid antigens are known to both enhance as well as suppress secretion of pro-inflammatory cytokines such as TNF-α, interleukin (IL)-1α, IL-1β, and IL-6 by host macrophages (Lee et al., 2007; Thirunavukkarasu et al., 2013, 2017b; Howard et al., 2018). However, the role of these cytokines in altering host lipid metabolism and promoting macrophage foam cell formation during *Mtb* infection has not been addressed. In contrast, both type I and type II interferon (IFN)-induced alterations in host lipid metabolism leading to either mycobacterial persistence or protective immunity has been previously addressed (Donovan et al., 2017; Knight et al., 2018). While type II signaling is associated with protective immunity and lipid droplet formation involving PGE2 and LXB4, type I (Knight et al., 2018), signaling suppresses IL-1β induced protective PGE2 conferring susceptibility to mycobacterial infection (Mayer-Barber and Yan, 2016).

Recently there has been a renewed interest in the role of type I interferons in affecting *de novo* cholesterol and fatty acid synthesis as well as influencing uptake of exogenous lipids by cells during the course of infectious diseases (Blanc et al., 2011, 2013; York et al., 2016). The role of type I IFN in specific cellular subsets is also being explored with recent reports claiming that pDCs (plasmacytoid dendritic cells) which characteristically produce copious type I IFN (Swiecki and Colonna, 2015) alter cellular metabolism by increasing fatty acid oxidation and oxidative phosphorylation, which is mediated in an autocrine manner by the type I IFN secreted (Wu et al., 2016). Moreover, type I IFN signaling via signal transducer and activator of transcription 1 (STAT-1) enhances expression of the cholesterol 25 hydroxylase (*ch25h)* gene and production of 25-hydroxy cholesterol as well as miR342-5p which suppresses the sterol biosynthetic pathway (Robertson et al., 2016). The lipophilic sterol metabolites produced by IFN signaling including oxysterols are substrates for Cyp enzymes and they profoundly influence cellular metabolism (Newmark et al., 2017) which in turn has been shown to influence *Mtb* replication inside cells (Huang et al., 2018). Since cytokine modulation of host-lipid metabolism seems to have a profound influence in mycobacterial persistance detailed studies to delineate the signaling mechanisms involved is recommended.

### TB Granuloma and Host Lipid Metabolism

During disease progression in TB, granuloma is formed locally in the affected lungs. While a a solid granuloma is often associated with efficient containment of *Mtb*, a caseous granuloma is believed to favor rupture and dissemination of bacteria that likely results in clinical TB symptoms (Ehlers and Schaible, 2012). However, the underlying pathology at the site of infection that determines progresssion to active TB is yet to be fully estalished. Research focused in identifying the cellular mechanisms within TB granuloma have determined that host genes for lipid sequestration, synthesis, and metabolism are upregulated in caseous pulmonary TB granulomas (Kim et al., 2010). Moreover, biochemical analysis of these caseous granulomas identified LDL-derived host lipids such as cholesterol, cholesteryl esters, lactosylceramides, and TAG (Kim et al., 2010). In a different perspective, it is also known that mycobacterial infection results in enhanced PPAR expression and resultant lipid droplet formation in the host macrophages (Almeida et al., 2012). This is believed to occur via the utilization of TAG resulting in the limiting of bacterial replication while promoting drug tolerance of *Mtb* inside foamy macrophages, which is a mechanism of disease perpetuation (Daniel et al., 2011). Moreover, *Mtb* is known to modulate the kinetics of cholesterol metabolism in host macrophages mainly by enhancing the expression of macrophage LXR (liver X receptor) genes, which eventually increases bacterial replication inside the foamy macrophages in granulomatous lesions (Vermeulen et al., 2017). In addition, the presence of an association between vitamin D receptor (VDR) and lipid metabolism in human tuberculosis and infected macrophages has also been reported (Salamon et al., 2014). When infected macrophages were treated with vitamin D, the accumulation of lipid droplets was abolished via a mechanism that involved VDR mediated downregulation of the proadipogenic PPARγ in infected macrophages. Also administration of PPARγ agonists reversed the antiadipogenic as well as the antimicrobial effects of VDR, thus establishing a role for vitamin D in macrophage lipid metabolism (Salamon et al., 2014).

Therefore, manipulation of the endogenous lipid mediators discussed above as well as cytokines involved in altering the lipid metabolic pathway of the host during mycobacterial infections has started to gain importance in studies addressing mycobacterial pathogenesis with the aim of identifying targets for vaccine development and drug discovery.

## Cardiovascular Disease to Mycobacterial Infection: Research Avenues to Extrapolate and Explore

Since macrophages are the primary cell type involved in foam cell formation in atherosclerosis and mycobacterial infections, the biogenesis of macrophage foam cell formation may likely be similar. While many of the receptors and enzymes involved in foam cell formation have been extensively researched in relation to CVD (Schumacher and Benndorf, 2017) their application in relation to mycobacterial pathogenicity is not yet fully understood.

### Lipid Transporters, Cytokines, Lipid Mediators, and Foam Cell Research

Since mycobacteria are intracellular pathogens often infecting macrophages, the contribution of macrophage foam cell formation to mycobacterial pathogenesis has been addressed. However, there are several areas which could benefit from the application of relevant knowledge available in the CVD field. For example, the ABC transport proteins not only play a role in lipid transport in host macrophages but in *Mtb* a total of 26 complete ABC transporters have been cataloged with many of the ABC exporters potentially implicated in the transport of drugs, probably contributing to the resistance of *Mtb* to many antibiotics (Braibant et al., 2000). While the role of mycobacterial ABC transport proteins and phospholipases has been explored (Braibant et al., 2000; Glass et al., 2017), the importance of host ABC proteins in mycobacterial diseases could be further explored. Recently Long et al. profiled the plasma membrane of macrophages infected with *Mtb* and reported an upregulation of ABCA1 which is involved in cholesterol efflux from the macrophages (Long et al., 2016). Similarly, few studies have reported on the importance of other transporters/receptors such as MARCO for induction of TNF-α, IL-1β, IL-6, and other responses to mycobacterial trehalose dimycolate and *Mtb* (Benard et al., 2014; Thuong et al., 2016; Khan et al., 2017). These avenues could be explored further in the context of TB disease reactivation.

IL-6 is a key cytokine induced by Angiotensin-II which is involved in the development of several CVDs including atherosclerosis (Schieffer et al., 2004; Kokje et al., 2016; Akita et al., 2017). IL-6 causes an increase in macrophage uptake of oxLDL via increase in expression of scavenger receptors such as SR-A, lectin-like OxLDL receptor-I, and CD36 (Keidar et al., 2001; Hashizume and Mihara, 2012). Mycobacteria, as well as their cell wall components, have been established to induce IL-6 production by varying cell types including macrophages, dendritic cells, and platelets (Adams and Czuprynski, 1994; Champsi et al., 1995; Jang et al., 2004). Apart from systemic production of IL-6, its expression locally at the granulomatous lesion has been reported by Renshaw et al. who have confirmed that thrombocytosis associated with *Mtb* infection in the granuloma was accompanied by elevated levels of IL-6 (Renshaw and Gould, 2013). Moreover, mycobacteria infected murine macrophages reportedly produce 10,000-fold more IL-6 compared to uninfected controls (Vanheyningen et al., 1997). Therefore, it would be worthwhile to assess how IL-6 is associated with mycobacteria-induced foam cell formation and *Mtb* survival in a non-replicative state.

Among the lipid mediators, isoprostanes and isoketals produced by lipid peroxidation of AA have been studied in detail in CVD pathogenesis (Cracowski and Ormezzano, 2004; Roberts and Milne, 2009; Kirabo et al., 2014). F2 isoprostanes containing oxidized phospholipids rapidly adduct to the amino group of lysine residue of cell membrane proteins disrupting their structural and functional capacity resulting in cellular dysfunction (Brame et al., 2004; Sullivan et al., 2010). Isoketals in particular are highly cytotoxic and their production is enhanced by oxidative stress (Kirabo et al., 2014). Since the immune response to mycobacterial infection is characterized by an oxidative burst, it is likely that these agents are generated within the inflammatory milieu during infection which might influence the disease progression and outcome, however their importance in TB has not been explored. Isoketals increase binding and uptake of LDL (Sullivan et al., 2010) and thus also promote foam cell formation which is an important criterion promoting mycobacterial survival. Moreover, isoketals have a role in DC activation by crosslinking lysine residues on proteins, altering protein function causing them to become antigenic (Brame et al., 2004; Kirabo et al., 2014) which might be a source of persistant uncontrolled inflammation aggravating pulmonary tissue destruction resulting in fibrotic development (Mont et al., 2016).

### Alternate Cell Types: Platelets

The importance of platelets in inflammation mediated diseases and disorders including atherosclerosis, arthritis, and cancer has now been established (Ferrer-Acosta et al., 2014; Papapanagiotou et al., 2016). Platelets have the capacity to facilitate inflammatory cell infiltration at the lesion site resulting in the release of a milieu of inflammatory mediators (Stokes and Granger, 2012). Moreover, in atherosclerosis LDL initiates a platelet-mediated signaling cascade and facilitates foam cell formation. Platelets initiate maturation of macrophages promoting internalization of oxLDL via scavenger receptors aiding in the formation of foam cells (Huo et al., 2003; Siegel-Axel et al., 2006; Von Hundelshausen and Weber, 2007; Von Hundelshausen and Schmitt, 2014). Interaction of platelets with monocytes results in an increase in the circulating population of CD14^hi^ CD16^+^ monocytes in humans (Passacquale et al., 2011; Tapp et al., 2011) and these are the subpopulations that generally transform to foam cells, as enzymatically degraded LDL preferentially binds to these cells via enhanced CD36 expression (Kapinsky et al., 2001). In a study by Huo et al. it was observed that injecting activated platelets resulted in the adhesion of monocytes to atherosclerotic lesions, leading to a reduction in the number of circulating monocytes, which suggests that platelet-monocyte adhesion plays a role in the formation of atherosclerotic lesions (Huo et al., 2003). Therefore, platelets are even being targeted for therapeutic development to reduce atherosclerosis and atherothrombosis (Lindemann et al., 2001, 2007).

One of the first studies assessing the direct involvement of platelets in bacterial pathogenesis was by Sullam et al. who reported through activation of the COX pathway and resultant production of thromboxane A2, platelets could directly kill *Streptococcus sanguis in vitro* (Sullam et al., 1993). Although the role of macrophages and T cells in protection against mycobacteria has been well-established (Ernst, 2012), the role of platelets in mycobacterial infections has lagged behind and the importance of these cells in relation to TB pathogenesis has gained priority only in the recent past (Lugo-Villarino and Neyrolles, 2014). Pulmonary TB due to *Mtb* infection is now associated with increased platelet infiltration and activity, coupled with enhanced platelet interaction with monocytes and T cells in granulomas, stressing a critical role for these cells in TB pathogenesis (Renshaw and Gould, 2013; Kullaya et al., 2018).

In atherosclerosis, apart from macrophages, other cell types including smooth muscle cells and endothelial cells lining the blood vessel can also transform into foam cells (Glukhova et al., 1987; Ivan and Antohe, 2010). Similarly, whether other cell types present locally at the site of granuloma formation eg. alveolar cells, airway smooth muscle cells (in *Mtb* infection) or intestinal smooth muscle cells (in *M. paratuberculosis* infection) could form foam cells and how that would drive the disease course has not been addressed. In this context the role of platelets in host lipid metabolism during mycobacterial infection was only recently addressed, wherein platelets were found to have the capacity to initiate the differentiation of monocytes into epithelioid-like multinucleated giant foam cells of the suppressive phenotype (Feng et al., 2014). However, other aspects of platelet-mediated signaling cascades and associated alterations in lipid metabolism including the activation state of platelets, their interaction with monocyte subsets and T cells driving foam cell formation and/or antigen presentation and adaptive immunity during mycobacterial infections are avenues which could be further explored utilizing literature available in depth in this area in CVD research as guidelines.

### Cytochrome P450 (Cyp) System

The Cyp system has been implicated in the pathogenesis of various CVDs including atherosclerosis and aneurysms (Song et al., 2016; Thirunavukkarasu et al., 2016b). The Cyp enzymes are heme containing monooxygenases involved in the pathways associated with the metabolism of fatty acids, steroids and other lipophilic molecules (Pikuleva, 2008). The loss of Cyp enzymes prevents Apolipoprotein (Apo) A1 synthesis thus affecting the removal of cholesterol from the cells to the liver for biliary excretion (Rubin et al., 1991; Nebert and Russell, 2002; Guan et al., 2003). The cholesterol metabolizing ability of Cyp family members varies depending on the physiological requirements of different organs and *in vivo* cholesterol levels. Since the activity of the Cyp enzymes can be modulated post-translationally, they are targets to manipulate cholesterol homeostasis (Luoma, 2008).

The importance of Cyp in mycobacteria is known. For example, *Mtb* has about 20 different Cyp enzymes, and several Cyp genes have been associated with mycobacterial viability (Mclean et al., 2010). Cole et al. have reported that the *Mtb* genome sequence has a very high number of Cyp enzymes, with Cyp128 contributing to synthesis of cell wall sulpholipid and being necessary for *in-vitro* growth of the bacterium (Cole et al., 1998; Holsclaw et al., 2008; Sogi et al., 2016). Iron is necessary for mycobacterial Cyp (Ouellet et al., 2002, 2011) activity and iron has been recognized as a crucial element for the survival of intracellular mycobacteria (Ratledge, 2004; Janagama et al., 2009; Lamont et al., 2013). Thus, the importance of Cyp from the pathogen perspective has been explored albeit not in depth.

From the host perspective, a transcriptomic analysis of TB granulomas has revealed a characteristic downregulation of several *Cyp* genes in cells within these granulomas (Eisenreich et al., 2013). Cyp monooxygenases are involved in biosynthesis of oxysterols (Pikuleva, 2006, 2008), however the dynamics between the expression of *Cyp* genes in granuloma formation and cholesterol metabolism has not been assessed in *Mtb* infection.

#### Cyp450 and Steroid Sex Hormones

Physiologically occurring steroid hormones which impact sexual characteristics are lipids that are synthesized from cholesterol in the gonads and adrenal glands. These sex steroid hormones have additional functions influencing metabolism, inflammation and immune responses. Apart from their direct role in modulating host cholesterol metabolism, members of the Cyp family such as Cyp4501b1(Cyp1b1) are involved in the metabolism of sex steroidal hormones with the resultant metabolites produced being implicated to play a role in the gender differences noticed to the susceptibility to cardiac and renal diseases (Jennings et al., 2014; Pingili et al., 2015, 2016; Song et al., 2016; Thirunavukkarasu et al., 2016b). While Cyp1b1 has been shown to have a protective role against cardiac and renal diseases in females, it aggravates the pathogenesis of these diseases in males, due to the action of this enzyme on estrogen and testosterone, respectively, as shown by ovariectomy and castration studies (Jennings et al., 2014; Pingili et al., 2016, 2017).

Sexual dimorphism in the relative susceptibility to *Mtb* and other mycobacterial infections has been reported, with the male to female ratio of susceptibility to *Mtb* infection reported to be 1.9/0.6 worldwide (Yamamoto et al., 1991; Neyrolles and Quintana-Murci, 2009; Karunasena et al., 2014; Nhamoyebonde and Leslie, 2014). Both in humans and animal models it has been shown that the male gender is more susceptible to TB than the female gender, and the differences in sex hormones could be a possible underlying mechanism for this difference (Svanberg, 1981; Tsuyuguchi et al., 2001; Ramsey et al., 2006; Dibbern et al., 2017). Intact female and castrated male mice have reduced severity of *M. avium, M. marinum*, and *M. intracellulare* infection, while ovariectomized females, or females and castrated males treated with testosterone had increased susceptibility to infection (Yamamoto et al., 1991; Tsuyuguchi et al., 2001). These findings have translational significance as in humans the death rate in castrated males due to TB was less compared to intact males and pre-menopausal females (Hamilton and Mestler, 1969). Moreover, there is almost a 2-fold increase in the severe lepromatous form of tuberculoid leprosy in males compared to females (Guerra-Silveira and Abad-Franch, 2013). These studies suggest that sex steroidal hormones might have a role in influencing the susceptibility to mycobacterial infections in mammals. Therefore, extrapolating from the evidence for Cyp1b1 in gender specific pathogeneis of CVDs, it would be worthwhile to explore further the role of Cyp family members in mycobacterial infections due to a necessity for a sex-tailored therapeutic approach in view of the gender related susceptibility to disease.

### Technology Transfer

3D *in vitro* technology employing scaffolds made of biomaterials that could be engineered to simulate tissue degeneration or regeneration has revolutionized biomedical research in CVD medicine (Ou and Hosseinkhani, 2014). Such models could be employed in mycobacterial research as well, to assess, understand, and decipher novel mechanisms involved in the pathogenesis of these bacteria in a dynamic sense. Positive steps in this direction were initiated by devising a three dimensional *in vitro* granuloma mode utilizing PBMCs to study host-pathogen interactions, drug susceptibility as well as mycobacterial drug tolerance and persistence (Kapoor et al., 2013; Fitzgerald et al., 2014). Since foam cell formation is associated with *Mtb* survival during infection research in *in vitro* models of *Mtb*-infected foam cells is critical. Novel methods to generate and evaluate foam cells *in vitro* have been utilized in the CVD research field (Xu et al., 2010; Sengupta et al., 2013), and these techniques could be applied to assess the interaction of mycobacteria with foam cells under controlled conditions to gain a better understanding of the events that lead to persistence of the bacterium in a non-replicative state as reported by Santucci et al. (2016). Thus, incorporating the recent advances in *in vitro* technology employed in CVD research to study foam cell signaling in mycobacterial research should prove fruitful.

## Conclusions

Decrease in cholesterol efflux, increase in uptake, and excess esterification of cholesterol compounded by the action of lipid peroxidation products generated by inflammatory processes contribute to foam cell formation in atherosclerosis. Since these are critical events in the pathogenesis of atherosclerosis, they have been extensively studied. On the contrary, although a role for host lipid metabolism in mycobacterial pathogenesis has been established, research in the different components that contribute to the alteration in host lipid metabolism and cholesterol homeostasis is still in the nascent stages. The specific cause for reactivation of mycobacterial disease from a controlled state in an infected host is still a critical question that remains inconclusive. It is speculated that the reversible lipid accumulation in foamy macrophages could be a possible phenomenon that is associated with disease reactivation (Caire-Brändli et al., 2014). Researchers attempting to decipher these loopholes in TB pathogenesis specifically targeting the host lipid pathway with the aim of developing novel therapeutics and vaccination strategies would be better served by utilizing the plethora of resources available in this area in the cardiovascular field (Kurth et al., 2004; Han et al., 2017; Rodriguez et al., 2017). The potential for PPAR regulators of airway inflammation as potential therapeutic targets for asthma and lung diseases (Standiford et al., 2005; Banno et al., 2018) has already been explored and their application for TB therapeutics could be further explored. Some studies have already reported on the application of statins and COX-2 inhibitors as adjuncts in the therapeutic strategy of mycobacterial infections which could be further exlpored in depth (Turull and Queralt, 2000; Brombacher et al., 2013; Lobato et al., 2014). Thus, extrapolation and application of relevant knowledge and techniques between these fields would prove to be beneficial and sustainable amidst an increasingly competitive research scenario beset by time and funding constraints.

## Author Contributions

ST performed the literature review and wrote the first draft of the manuscript. SK critically revised the paper. All authors read and approved the final version of the manuscript.

### Conflict of Interest Statement

The authors declare that the research was conducted in the absence of any commercial or financial relationships that could be construed as a potential conflict of interest.

## Abbreviations

CVD: cardiovascular disease
TB: tuberculosis
*Mtb*: *Mycobacterium tuberculosis*
AA: arachidonic acid
COX: cyclooxygenase
LOX: lipoxygenase
CYP: Cytochrome
oxLDL: oxidized low density lipoprotein
PGE2: Prostaglandin E2
IFN: interferon.
